# Cardiovascular and respiratory effects of lumbosacral epidural bupivacaine in isoflurane-anesthetized dogs: The effects of two volumes of 0.25% solution

**DOI:** 10.1371/journal.pone.0195867

**Published:** 2018-04-18

**Authors:** Raquel Sartori Gonçalves Dias, João Henrique Neves Soares, Douglas dos Santos e Castro, Maria Alice Kuster de Albuquerque Gress, Marcela Lemos Machado, Pablo E. Otero, Fabio Otero Ascoli

**Affiliations:** 1 Graduate Program in Cardiovascular Sciences, College of Medicine, Fluminense Federal University (UFF), Niterói, Rio de Janeiro, Brazil; 2 Department of Small Animal Clinical Sciences, Virginia–Maryland Regional College of Veterinary Medicine, Virginia Tech, Blacksburg, Virginia, United States of America; 3 Department of Large Animal Clinical Sciences, Anesthesia and Pain Management Service, University of Florida, Gainesville, Florida, United States of America; 4 Laboratory of Animal Research, Veterinary School, Fluminense Federal University, Niteroi, Rio de Janeiro, Brazil; 5 Universidad de Buenos Aires, Facultad de Ciencias Veterinarias, Cátedra de Anestesiología y Algiología, Buenos Aires, Argentina; 6 Department of Physiology and Pharmacology, Biomedical Institute, Fluminense Federal University, Niterói, Rio de Janeiro, Brazil; University of Bari, ITALY

## Abstract

The purpose of this study was to compare cardiovascular and respiratory effects of two volumes of bupivacaine 0.25% (0.2 mL kg^-1^—treatment BUP02—and 0.4 mL kg^-1^ –treatment BUP04) administered epidurally at the lumbosacral intervertebral space in dogs anesthetized with isoflurane. This experimental prospective randomized crossover design trial used six mixed breed adult dogs, four neutered males and two spayed females. Each dog was anesthetized on three different occasions: the first for isoflurane minimum alveolar concentration (MAC) measurement, and the following two assigned treatments (BUP02 or BUP04). On the two treatment days, anesthesia was induced and maintained with isoflurane at 1.3 MAC during the experiments. Cardiovascular and respiratory measurements were recorded before (T0) and 5, 15, 30, 60 and 90 minutes after the epidural administration of bupivacaine. Comparisons between and within groups were performed by a mixed-model ANOVA and Friedman’s test when appropriate followed by Bonferroni post-hoc test or Dunnet’s test to compare time points within each treatment with T0 (*p* < 0.05). Mean arterial pressure decreased significantly from 15 to 90 minutes after the administration of BUP02 and from 5 to 60 minutes in BUP04, with lower values in BUP04 than in BUP02 lasting up to 30 minutes after bupivacaine administration. No significant changes in cardiac output and systemic vascular resistance were observed in either treatment. Hypoventilation was only detected in BUP04. Hemoglobin concentration and arterial oxygen content decreased after both treatment of bupivacaine with no significant decrease in oxygen delivery. Two dogs in BUP04 developed Horner’s syndrome. The epidural administration of 0.4 mL.kg^-1^ of bupivacaine to dogs in sternal recumbency anesthetized with isoflurane 1.3 MAC caused more cardiovascular and respiratory depression than 0.2 mL.kg^-1^.

## Introduction

Epidural anesthesia has been widely used as an adjuvant anesthetic technique in dogs due to its perioperative analgesia [1], reduction of general anesthetics requirements [2, 3], muscle relaxation, and attenuation of the stress response to surgery [4, 5]. Epidural anesthesia has been associated with improved clinical outcome in humans, but this association has not yet been demonstrated in dogs [6, 7]. The lumbosacral space is the most common site for epidural anesthesia in dogs, and bupivacaine is one of the most commonly used local anesthetic for this technique [8]. Epidural anesthesia is commonly combined with inhalant anesthetics in canine surgical patients [1,8].

Despite the benefits mentioned above, adverse cardiovascular and respiratory effects can be associated with the use of epidural administration of anesthetics [3, 9–13]. This is due to the blocking effect on motor and autonomic neurons. These effects can vary in duration and intensity depending on volume and concentration of the anesthetic that is used [13, 14].

A volume of solution that is often recommended for epidural anesthesia in dogs is 0.2 mL.kg^-1^ [1, 8]. Nevertheless, the volume of epidural bupivacaine 0.25% positively correlates with the desensitization of more cranial dermatomes [15]. This property of epidural local anesthetics may be desirable for upper abdominal and thoracic surgeries. A volume of 0.36 mL.kg^-1^ of bupivacaine has been recommended for epidural anesthesia in ovariohysterectomies in dogs [16], since the ovarian nociceptive pathways converge to high lumbar and low thoracic spinal nerves [8, 17]. Nevertheless, the epidural administration of a high volume of local anesthetics may induce remarkable adverse hemodynamic and respiratory effects due to a more cranial and extended autonomic and motor blockade regardless of the concentration and the local anesthetic investigated [11–13, 18]. In addition, Horner’s syndrome has been observed in patients that presented with high thoracic (T1-T3) sympathetic block [11, 12, 15, 19].

The epidural administration of bupivacaine decreases arterial blood pressure by decreasing cardiac output (CO) and/or systemic vascular resistance (SVR). However, the pathophysiology of hypotension due to the epidural administration of bupivacaine seems to depend on its spinal cord level in conjunction with the extent of sympathetic blockade [20–22], and on the presence of other drugs such as inhalant anesthetics [23, 24]. The sympathetic blockade from epidural local anesthetics at the lumbar spinal level causes vasodilation and a decrease in SVR, while the decrease in CO due to decrease in heart rate and contractility is only seen when the local anesthetics migrate to higher thoracic levels (T1 –T5) and block the cardiac accelerator nerves [8, 13]. The thoracic administration of 1 mL of bupivacaine 0.5% caused a significant decrease in myocardial contractility in pigs (36 kg) anesthetized with propofol and sufentanil, while the lumbar epidural administration of 4 mL caused a marked decrease in SVR. A decrease in mean arterial pressure (MAP) was observed in both treatments but was more pronounced in the lumbar group with no change in CO [20]. In awake dogs, approximately 0.25 mL kg^-1^ of lumbar epidural bupivacaine 0.75% caused significant decrease in CO and MAP with no apparent effect on SVR [21]. By contrast, 0.5 to 0.6 mL kg^-1^ of bupivacaine 0.5% administered through an epidural catheter with its tip localized in the high lumbar/low thoracic region caused significant decreases in MAP, CO, SVR in thiopental/nitrous oxide anesthetized dogs [22]. The incidence of hypotension in dogs was eight times higher when bupivacaine was added to morphine in the epidural treatment of dogs undergoing orthopedic surgeries [25].

Respiratory depression from epidural anesthesia seems to be mainly caused by motor block at the thoracic level impairing the function of intercostal muscles and at the cervical level (C4-C7) blocking the phrenic nerve and the diaphragm function [12, 18]. Respiratory depression was observed in awake dogs after the administration of 0.6 to 0.8 mL kg^-1^ and severe hypoventilation was developed in isoflurane-anesthetized dogs after the administration of 0.6 mL kg^-1^ of 0.25% bupivacaine [11]. In addition, the cardiovascular depression promoted by 0.6 mL.kg^-1^ was potentiated by the hypoventilation [11].

Bupivacaine is clinically available in three different concentrations– 0.25, 0.5 and 0.75%. For a constant volume of epidural bupivacaine, a lower concentration results in analgesia and motor block which is less intense and shorter in duration [14]. The same principles can probably be applied to the cardiovascular and respiratory effects, and because of this, comparisons between studies with different bupivacaine concentrations should be critically evaluated. To the authors’ knowledge, there is no study evaluating the cardiovascular and respiratory effects of the epidural administration of bupivacaine 0.25% in isoflurane anesthetized dogs, particularly when different volumes of solution are compared.

The purpose of this study was to compare the cardiovascular and respiratory effects of two volumes (0.2 and 0.4 mL kg^-1^) of epidural 0.25% bupivacaine administered in spontaneously breathing dogs anesthetized with 1.3 MAC of isoflurane. The hypothesis of the present study was that 0.2 mL kg^-1^ of bupivacaine 0.25% would cause less cardiovascular and respiratory depression than 0.4 mL kg^-1^.

## Material and method

### Animals

The Institutional Animal Care and Use Committee of Universidade Federal Fluminense approved the protocol described in this manuscript (n.o 0098/2011). Six healthy adult mixed-breed dogs (4 neutered males and 2 spayed females) weighing 18.9 ± 3.3 kg (mean ± SD) were used in this study. The dogs had free access to dry food (PremieR Pet, SP, Brazil) and water. Health status was based on physical examination, complete blood cell count, and serum biochemistry analysis. This study was performed on three experimental days for each dog with at least a one-week interval between experiments. On the first experimental day, the dogs were anesthetized to have their individual isoflurane MAC determined, and on the second and third day the cardiovascular and respiratory effects of each volume of epidural bupivacaine (0.2 or 0.4 mL kg^-1^) were evaluated. On all experimental days, food was withheld from each dog 12 hours prior to the experiment.

### Instrumentation common to all experimental days

Anesthesia was induced with isoflurane dialed at 5% on the agent-specific vaporizer (Sigma Delta, Penlon Ltd., OX, UK) and delivered by a facemask with an oxygen flow of 5 L min^-1^ using a circle breathing system. After endotracheal intubation, the endotracheal tube was connected to the circle breathing system and isoflurane (Cristália Produtos Químicos Farmacêuticos Ltda, SP, Brazil) was delivered in oxygen at a rate of 50 mL kg^-1^ minute. The dog was spontaneously ventilating in lateral recumbency with an end-expiratory isoflurane concentration (FE´ISO) at approximately 2% during instrumentation. A cephalic intravenous catheter was placed for administration of Lactated Ringer’s Solution at a rate of 3 mL kg^-1^ hour^-1^, and an arterial catheter was placed in the dorsal pedal artery to measure systolic (SAP), mean (MAP) and diastolic (DAP) pressure and to collect blood samples for gas analysis. All pressure transducers used in this study were calibrated against a mercury column before each experiment and their zero reference was set at the level of the sternum manubrium in each dog.

Body temperature was maintained between 37.5 and 38.5°C by means of an electrical heating pad (Brasmed Veterinária, SP, Brazil). Pulse rate (PR) was measured from the arterial pressure waveform in the absence of artifacts (LW6000 –Digicare Biomedical Technology, FL, USA). Respiratory rate (*f*_R_) was measured by capnography always in the presence of a regular respiratory rhythm with no artifact on the capnograms. In the presence of artifact in blood pressure and capnogram, PR and *f*_R_ were counted over a minute. End-expiratory carbon dioxide tension (PE´CO2) and FE´ISO were measured by infrared technique (LW6000 –Digicare Biomedical Technology, FL, USA). The gas analyzer was verified before the experiments by the internal electronic protocol provided by the manufacturer.

### MAC determination

Isoflurane MAC was determined following a bracketing technique by using a square-wave electrical noxious stimulus of 30 mA and 50 Hz applied to the cranial aspect of the tarsus by two stainless steel needles subcutaneously positioned approximately 3 cm apart [26]. This noxious stimulation was sustained for 60 seconds or until a positive response was observed. Movements of the stimulated limb, swallowing, blinking or increased respiratory effort were not considered positive responses. Any movement of other body parts (mainly the head or limbs) in response to the noxious stimulation was considered a positive response. FE´ISO was increased or decreased by 10 to 20% of the previous concentration after a positive or negative response, respectively, and 15 minutes were allowed for equilibration at each step before another noxious stimulation. MAC was calculated as the average of two consecutive FE´ISO—one positive and one negative (or vice versa). MAC was determined twice in each dog and the average was reported as the final MAC. Meloxicam 0.2 mg/kg was administered intravenously immediately after finishing MAC determination. At this point, isoflurane delivery was stopped and the dogs were allowed to recover from anesthesia. The dogs were returned to their kennel after reaching a rectal temperature between 37.5 and 38.5°C and normal ambulation.

### Cardiovascular and respiratory measurements and calculations

After the initial instrumentation, an 8 F introducer sheath (Arrow International, Inc., PA, USA) was placed in the right jugular vein followed by the introduction of a 7 F pulmonary artery (PA) catheter (Edwards Lifesciences LLC, CA, USA). The tip of this catheter was confirmed to be in the PA by visualization of typical waveforms of pulmonary artery pressure (PAP) and pulmonary artery occlusion pressure (PAoP) displayed on the monitor (DX 2021; Philips Healthcare, Netherlands). The PA catheter was used to measure cardiac output (CO), central venous pressure (CVP), PAP, PAoP, and core temperature, as well as to obtain mixed venous samples from the PA. Intermittent measurements of PAoP were obtained by inflating the balloon located at the tip of the PA catheter with 0.7 ml of air. Cardiac output was measured by the rapid injection of 5 mL of cold saline solution (0–5°C) at the beginning of expiration and was reported as the average of three consecutive measurements within 10% of each other.

Tidal volume (V_T_) was measured by the ventilator’s differential pressure fixed orifice pneumotachometer, which was verified by the ventilator protocol (SAT500, KTK, SP, Brazil) at the beginning of the experiments. The protocol of breathing system compliance compensation was also performed before the experiments and minute ventilation (${\dot{\text{V}}}_{\text{E}}$) was calculated as V_T_ x *f*_R_. Temperature-corrected measurements of arterial and mixed venous blood pH, partial pressures of oxygen and carbon dioxide, as well as the calculation of bicarbonate concentration (HCO_3_^-^), base excess with no correction for hemoglobin (BE) and hemoglobin oxygen saturation were immediately obtained with a portable blood gas analyzer (I-STAT—Abbott, IL, USA).

The calculations used in this study followed standard formulas and are described below:

$$\text{Body}\;\text{surface}\;\text{area}\;\left({\text{BSA-m}}^{\text{2}}\right)\;=\;0.101\times BW^{0.67},$$

where BW is body weight in kg;

$$\text{Cardiac}\;\text{Index}\;\left({\text{CI-L}\;\text{min}}^{\text{-1}}{\text{m}}^{\text{-2}}\right)\;=\;\frac{CO}{BSA};$$

$$\text{Stroke}\;\text{Index}\;\left({\text{SI-mL}\;\text{beat}}^{\text{-1}}{\text{kg}}^{\text{-1}}\right)\;=\;\frac{CO}{PR\times BW};$$

$$\text{Systemic}\;\text{vascular}\;\text{resistance}\;\text{index}\;\left(\text{SVRI-dyne}\;\text{second}\;{\text{cm}}^{\text{-5}}{\text{m}}^{\text{-2}}\right)\;=\;80\;\times \frac{MAP-CVP}{CI};$$

$$\text{Pulmonary}\;\text{vascular}\;\text{resistance}\;\text{index}\;\left(\text{PVRI}-\text{dyne}\;\text{second}\;{\text{cm}}^{-5}{\text{m}}^{\text{-2}}\right)\;=\;80\;\times \frac{\text{PAP}-\text{PAoP}}{\text{CI}}\text{;}$$

$$\text{Left}\;\text{ventricular}\;\text{stroke}\;\text{work}\;\text{index}\;\left(\text{LVSWI}-\text{cJ}\;{\text{kg}}^{-1}\right)\;=\;0.0136\;\times \left(\text{MAP}-\text{PAoP}\right)\;\times \;\text{SI;}$$

$$\text{Right}\;\text{ventricular}\;\text{stroke}\;\text{work}\;\text{index}\;\left({\text{RVSWI-cJ}\;\text{kg}}^{\text{-1}}\right)\;=\;0.0136\;\times \left(\text{PAP-CVP}\right)\;\times \;\text{SI};$$

$$\text{Arterial}\;\text{oxygen}\;\text{content}\;\left({\text{CaO}}_{\text{2}}{\text{-mL}\;\text{dL}}^{\text{-1}}\right)=\left(1.39\;\times \;{\text{Hba}\;\times \;\text{SaO}}_{\text{2}}\right)+\left(\text{0}{.003\;\times \;\text{PaO}}_{\text{2}}\right),$$

where Hba is arterial hemoglobin concentration, SaO_2_ is oxygen saturation in the hemoglobin of arterial blood, and PaO_2_ is the arterial partial pressure of oxygen;

$$\text{Mixed}\;\text{venous}\;\text{oxygen}\;\text{content}\;\left({\text{C}\bar{\text{v}}\text{O}}_{2}{\text{-mL}\;\text{dL}}^{\text{-1}}\right)\;=\;\left(1.39\;\times \;\text{Hb}\bar{\text{v}}\;\times \;{\text{S}\bar{\text{v}}\text{O}}_{2}\right)+\left(0.003\;\times \;{\text{P}\bar{\text{v}}\text{O}}_{2}\right),$$

where $\;\text{Hb}\overset{-}{\text{v}}\;$ is mixed venous hemoglobin concentration, $\text{S}\overset{-}{\text{v}}{\text{O}}_{\text{2}}$ is oxygen saturation in the mixed venous blood, and $\text{P}\overset{-}{\text{v}}{\text{O}}_{\text{2}}$ is the mixed venous partial pressure of oxygen.

$$\text{Oxygen}\;\text{Delivery}\;\text{Index}\;\left({\text{DO}}_{\text{2}}\text{I}-\text{mL}\;{\text{min}}^{\text{-1}}{\text{m}}^{\text{-2}}\right){\;=\;\text{CI}\;\times \;10\;\times \;\text{CaO}}_{\text{2}}\text{;}$$

$$\text{Oxygen}\;\text{Consumption}\;\text{Index}\left({\text{VO}}_{\text{2}}\text{I}-\text{mL}{\text{min}}^{\text{-1}}{\text{m}}^{\text{-2}}\right)=\text{CI}\times 10\times ({\text{CaO}}_{\text{2}}-{\text{C}\bar{\text{v}}\text{O}}_{\text{2}});$$

$$\text{Oxygen}\;\text{Extraction}\;\text{Ratio}\;\left({\text{O}}_{2}\text{ER}\right)\;=\;\frac{{\text{VO}}_{\text{2}}\text{I}}{{\text{DO}}_{\text{2}}\text{I}}$$

### Experimental protocol

After instrumentation, FE´ISO was adjusted to 1.3 MAC. Each dog was anesthetized twice with at least a one-week washout interval in a randomized crossover design for each volume of epidural bupivacaine 0.25%: 0.2 mL kg^-1^ (BUP02) or 0.4 mL kg^-1^ (BUP04). Randomization was achieved at the first day of experiment of each dog by choosing the treatment from a sealed opaque envelope.

The dogs were placed in sternal recumbency with the pelvic limbs extended cranially. Baseline (T0) cardiovascular and respiratory data were collected after at least 15 minutes of a steady FE´ISO at 1.3 MAC. Subsequently, the lumbosacral area was clipped and aseptically prepared. The lumbosacral intervertebral space was localized and an 18 gauge Tuohy needle (Becton Dickinson & Co., NJ, USA) was slowly introduced into the epidural space with its bevel directed towards the head until a “pop” sensation was felt. The correct position of the needle in the epidural space was confirmed by the lack of resistance to the injection of 0.5 to 1.0 mL of air using a glass syringe and the absence of blood and cerebrospinal fluid at the hub of the needle, as well as by the presence of the epidural pressure waveform when the epidural needle was connected to a fluid filled noncompliant arterial line and calibrated pressure transducer [27]. The syringe with bupivacaine was connected to the needle and an aspiration test was performed to confirm that the tip of the needle was not in a vessel. After confirmation that the needle was not in a vessel, epidural administration of bupivacaine 0.25% (Cristália Produtos Químico e Farmacêuticos Ltda, SP, Brazil) was performed over 2 minutes.

Cardiovascular and respiratory variables were recorded at T0, 5 (T5), 15 (T15), 30 (T30), 60 (T60) and 90 minutes (T90), after the epidural injection. Relative increases or decreases in MAP and its components CI and SVRI were calculated at each time point. Arterial and mixed-venous blood samples were anaerobically and simultaneously collected in non-heparinized syringes at T0, T15 and T60 for immediate blood gas analysis. The FE’ISO recorded during the experiments were recorded as multiples of each individual MAC.

Hypoventilation was defined as PE´CO2 higher than 45 mmHg. If PE´CO2 was higher than 60 mmHg, mechanical ventilation was initiated and maintained until the end of the experiment with V_T_ of 12 mL kg^-1^ and *f*r adjusted to maintain PE´CO2 between 35 and 45 mmHg. Mild hypotension was defined as MAP lower than 60 mmHg but higher or equal to 50 mmHg. If moderate hypotension (MAP < 50 and > 40 mmHg) occurred in the presence of PE´CO2 > 50 mmHg, mechanical ventilation was initiated. If MAP reached values lower than 50 mmHg in the absence of hypoventilation or after starting mechanical ventilation, dopamine (5 to 10 μg kg^-1^ minute^-1^) was started in order to maintain MAP higher than 50 mmHg. The incidence of mild and moderate hypotension was recorded during the experiments. Data from the dogs that were mechanically ventilated were excluded from the tables and statistical analysis.

### Post-anesthetic assessment

After the data collection at T90, 0.2 mg/kg of meloxicam (Ourofino Saúde Animal, SP, Brazil) was intravenously administered and the PA and arterial catheters were removed with manual compression applied in each dog until adequate hemostasis. Subsequently, the isoflurane vaporizer was turned off and the dogs were allowed to recover. Gross signs of motor dysfunction such as the inability to stand up and walk or any other significant residual effect from the epidural treatments were monitored and recorded for four hours after extubation. The dogs were returned to their kennel after removing the cephalic venous catheter and normal ambulation was observed.

### Statistical analysis

All statistical analysis was performed using the SAS University Edition 3.5 (SAS Institute Inc., NC, USA) and MatLab (MatLab 2015b, The Mathworks Inc., MA, USA). Normality of the data distribution was evaluated by the Shapiro-Wilk test and by visual inspection of normal probability plots. Normally and not normally distributed data were expressed as mean ± SD and median and interquartile range, respectively. The primary outcomes of this study were the main cardiovascular and respiratory parameters: MAP, CI, PR, SVRI, PaO_2_, PaCO_2_ and DO_2_I. For the normally distributed data, comparisons between treatments and within each treatment were performed by a mixed-model ANOVA having the dog as random effect and time point as repeated effect, while the Friedman’s test was used for the non-normally distributed data. T-test and Wilcoxon sum rank test were used to compare normally and non-normally distributed data, respectively, between the treatments within each time point. Bonferroni procedure was used to correct the p value for multiple comparisons. In addition, the Dunnet’s test was used to compare each time point within the treatments with their respective T0. p < 0.05 was considered sufficient to reject the null hypothesis.

## Results

The isoflurane MAC measured in the dogs of this study was 1.56 ± 0.18%. The FE´ISO recorded during each time point of evaluation is presented in Table 1 as multiples of MAC. No significant difference was found between and within the treatments.

**Table 1:** End-expiratory concentration of isoflurane (FE´ISO) expressed as multiples of minimum alveolar concentration before and after epidural administration of bupivacaine 0.25% in six dogs.

	GROUP	T0	T5	T15	T30	T60	T90
FE´ISO (%)	BUP02	1.30 ± 0.02	1.30 ± 0.01	1.29 ± 0.03	1.30 ± 0.03	1.31 ± 0.03	1.31 ± 0.03
	BUP04	1.32 ± 0.03	1.33 ± 0.09	1.33 ± 0.07	1.35 ± 0.06	1.34 ± 0.05	1.31 ± 0.05

T0 = baseline; T5 = 5 minutes after epidural administration; T15 = 15 minutes after epidural administration; T30 = 30 minutes after epidural administration; T60 = 60 minutes after epidural administration; T90 = 90 minutes after epidural administration; BUP02 = bupivacaine 0.25% at 0.2 mL kg^-1^; BUP04 = bupivacaine 0.25% at 0.4 mL kg^-1^.

In all animals the epidural pressure waveform was visualized and a positive “loss of resistance” test was observed. No blood or cerebrospinal fluid was aspirated into the needle prior to the epidural injection in any of the dogs.

Cardiovascular and ventilatory function, acid-base status, and oxygenation are reported in Tables 2, 3 and 4, respectively. There was a statistically significant difference between T0 and the other time points for MAP in both treatments (p < 0.0001), LVSWI in BUP04 (p = 0.027), PAP in both treatments (p = 0.0029), RVSWI in BUP04 (p = 0.027), V_T_ in BUP04 (p = 0.004), *f*_R_ in BUP04 (p = 0.0318), ${\dot{\text{V}}}_{\text{E}}$ in BUP04 (p < 0.0001), PaCO_2_ in BUP04 (p = 0.0183), BE in BUP04 (p = 0.0378), Hba in both treatments (p = 0.0017), Hbv in both treatments (p = 0.0017), CaO_2_ (p < 0.0001) in both treatments, and $\text{C}\overset{-}{\text{v}}{\text{O}}_{\text{2}}$ (p < 0.0001) in both treatments. In addition, there was a significant difference observed between groups for MAP (p = 0.0046), PAP (p = 0.0006), V_T_ (p = 0.00147), ${\dot{\text{V}}}_{\text{E}}$ (p = 0.0165), PaCO_2_ (p = 0.0318), and HCO_3_^-^ (p = 0.0318).

**Table 2:** Cardiovascular effects of two volumes of epidural bupivacaine (0.25%) in six dogs anesthetized with 1.3 minimum alveolar concentration of isoflurane.

Variables	Treatment	T0	T5	T15	T30	T60	T90
PR (beats minute^-1^)	BUP02	137 [135 145]	137 [122 139]	129 [115 138]	129 [116 144]	132 [118 148]	133 [120 142]
	BUP04^a^	125 [122 132]	117 [116 120]	114 [109 119]	118 [113 121]	123 [119 123]	124 [120 125]
MAP (mmHg)	BUP02	92 ± 15	80 ± 14	77 ± 16*	79 ± 17*	79 ± 12*	81 ± 14*
	BUP04^a^	86± 15	70 ± 8*†	58 ± 9*†	67 ± 3*†	72 ± 8*	78 ± 11
CVP (mmHg)	BUP02	5 ± 1	4 ± 2	4 ± 2	4 ± 2	4 ± 2	4 ± 2
	BUP04 ^a^	4 ± 2	4 ± 2	3 ± 1	3 ± 2	3 ± 1	3 ± 2
CI (mL minute^-1^ m^-2^)	BUP02	4.77 ± 1.82	4.14 ± 1.92	4.01 ± 2.11	4.34 ± 2.22	4.88 ± 2.75	4.89 ± 2.39
	BUP04^a^	4.50 ± 1.41	3.66 ± 1.00	3.84 ± 0.63	4.33 ± 0.47	4.70 ± 0.82	4.83 ± 0.82
SI (mL beat^-1^ kg^-1^)	BUP02	1.31 ± 0.43	1.19 ± 0.47	1.18 ± 0.52	1.24 ± 0.49	1.35 ± 0.61	1.37 ± 0.48
	BUP04^a^	1.38 ± 0.39	1.08 ± 0.33	1.15 ± 0.36	1.29 ± 0.40	1.38 ± 0.49	1.41 ± 0.51
SVRI (dynes second cm^-5^ m^-2^)	BUP02	1591 ± 474	1622 ± 453	1648 ± 528	1570 ± 510	1463 ± 538	1369 ± 321
	BUP04^a^	1606 ± 604	1553 ± 475	1167 ± 113	1192 ± 135	1179 ± 80	1254 ± 103
LVSWI (cJ kg^-1^)	BUP02	1.49 ± 0.69	1.21 ± 0.72	1.16 ± 0.81	1.25 ± 0.76	1.35 ± 0.83	1.39 ± 0.78
	BUP04^a^	1.43 ± 0.48	0.90 ± 0.32*	0.79 ± 0.32*	1.03 ± 0.32*	1.20 ± 0.49	1.36 ± 0.62
PAP (mmHg)	BUP02	19 ± 2	17 ± 2	16 ± 2*	16 ± 3	17 ± 2	17 ± 3
	BUP04^a^	17 ± 2	15 ± 1*	14 ± 2*	14 ± 2	14 ± 2	15 ± 1
PAoP (mmHg)	BUP02	11 [10 13]	9 [8 11]	9 [7 12]	9 [7 11]	9 [8 11]	10 [8 12]
	BUP04^a^	10 [8 12]	9 [8 10]	8 [7 9]	7 [6 9]	7 [6 10]	7 [6 9]
PVRI (dynes second cm^-5^ m^-2^)	BUP02	135 ± 39	167 ± 64	155 ± 71	157 ± 63	146 ± 57	132 ± 53
	BUP04^a^	123 ± 40	127 ± 42	120 ± 37	121 ± 48	102 ± 51	113 ± 44
RVSWI (cJ kg^-1^)	BUP02	0.26 ± 0.12	0.22 ± 0.12	0.20 ± 0.12	0.21 ± 0.12	0.25 ± 0.15	0.25 ± 0.15
	BUP04^a^	0.25 ± 0.09	0.17 ± 0.07*	0.18 ± 0.06*	0.20 ± 0.07	0.21 ± 0.09	0.25 ± 0.11

Data are expressed as mean ± SD, or median [interquartile range]. PR = Pulse rate; MAP = mean arterial pressure; CVP = central venous pressure; CI = cardiac index; SI = stroke index; SVRI = systemic vascular resistance index; LVSWI = left ventricle stroke work index; RVSWI = right ventricle stroke work index; PAP = pulmonary mean arterial pressure; PAoP = Pulmonary artery occlusion pressure; PVRI = pulmonary vascular resistance index.

^a^ = data shown for only 4 dogs

* = Significant difference between T0;

^†^ = significant difference between BUP02 and BUP04. p < 0.05

**Table 3:** Ventilatory function and acid-base status effects of two volumes of epidural bupivacaine 0.25% in six dogs anesthetized with 1.3 minimum alveolar concentration of isoflurane.

Variables	Treatment	T0	T5	T15	T30	T60	T90
*f*_R_ (breaths minute^-1^)	BUP02	15 ± 2	13 ± 2	12 ± 2	14 ± 2	14 ± 3	13 ± 3
	BUP04^a^	17 ± 5	10 ± 2*	10 ± 2*	12 ± 1*	14 ± 2	16 ± 4
V_T_ (mL kg^-1^)	BUP02	13.3 ± 3.9	13.1 ± 3.1	13.8 ± 2.4	13.7 ± 3.1	14.6 ± 2.5	15.1 ± 1.3
	BUP04^a^	12.8 ± 2.9	10.8 ± 3.0†	9.6 ± 2.3*†	11.4 ± 2.3*†	13.5 ± 2.7	14.8 ± 3.3
${\dot{\text{V}}}_{\text{E}}$ (mL kg^-1^ minute^-1^)	BUP02	202 ± 87	169 ± 37	161 ± 29	178 ± 45	193 ± 59	188 ± 48
	BUP04^a^	225 ± 95	104 ± 21*†	100 ± 36*†	140 ± 31*†	195 ± 60	233 ± 91
pH	BUP02	7.274 [7.236 7.297]	-	7.282 [7.239 7.300]	-	7.293 [7.253 7.315]	-
	BUP04^a^	7.280 [7.250 7.315]	-	7.203 [7.123 7.291]	-	7.290 [7.265 7.303]	-
PaCO_2_ (mmHg)	BUP02	54.0 ± 3.9	-	54.3 ± 6.0	-	54.0 ± 6.5	-
	BUP04^a^	48.0 ± 5.1†	-	62.6 ± 9.2*†	-	55.1 ± 1.5*	-
HCO_3_^-^ (mmol L^-1^)	BUP02	25.1 [25.0 25.3]	-	24.7 [23.8 25.4]	-	25.3 [24.7 25.9]	-
	BUP04^a^	23.2† [21.3 23.5]	-	22.0 [20.0 24.2]	-	24.8 [22.8 26.8]	-
BE (mmol L^-1^)	BUP02	-1.8 ± 1.5	-	-2.3 ± 1.5	-	-1.2 ± 1.3	-
	BUP04^a^	-2.2 ± 2.2	-	-3.3 ± 1.9	-	-0.8 ± 3.0*	-

*f*_R_ = respiratory rate; V_T_ = tidal volume; $\text{V}\dot{\text{E}}=\text{minute}\;\text{ventilation}$; PaCO_2_ = arterial partial pressure of carbon dioxide; HCO_3_^-^ = bicarbonate concentration in arterial blood; BE = base excess in the arterial blood.

^a^ = data shown for only 4 dogs;

* = Significant difference between T0;

^†^ = significant difference between BUP02 and BUP04. p < 0.05.

**Table 4:** Oxygenation parameters of two volumes of epidural bupivacaine 0.25% administered to six dogs anesthetized with 1.3 minimum alveolar concentration of isoflurane.

Variables	Treatment	T0	T15	T60
PaO_2_ (mmHg)	BUP02	527 ± 31	539 ± 26	542 ± 37
	BUP04^a^	536 ± 71	531 ± 24	531 ± 53
Hba (g dL^-1^)	BUP02	12.3 ± 1.0	11.2 ± 0.7*	11.3 ± 0.9*
	BUP04^a^	11.7 ± 0.5	10.7 ± 1.4*	10.8 ± 1.1*
CaO_2_ (mL dL^-1^)	BUP02	18.3 ± 1.3	16.9 ± 1.4*	17.2 ± 1.2*
	BUP04^a^	17.6 ± 0.6	16.4 ± 2.0*	17.0 ± 1.4
DO_2_I (mL minute^-1^ m^2^)	BUP02	879 ± 371	735 ± 394	832 ± 490
	BUP04^a^	796 ± 258	637 ± 162	806 ± 190
VO_2_I (mL minute^-1^ m^2^)	BUP02	85 ± 45	82 ± 19	106 ± 12
	BUP04^a^	97 ± 37	99 ± 7	124 ± 33
O_2_ER	BUP02	0.12 ± 0.08	0.14 ± 0.06	0.16 ± 0.07
	BUP04^a^	0.14 ± 0.08	0.16 ± 0.04	0.16 ± 0.03
$\text{P}\overset{-}{\text{v}}{\text{O}}_{\text{2}}$ (mmHg)	BUP02	89 [78 108]	72 [65 100]	74 [65 91]
	BUP04^a^	76 [65 121]	82 [73 91]	88 [82 92]
$\text{S}\overset{-}{\text{v}}{\text{O}}_{\text{2}}$ (%)	BUP02	93.3 ± 6.7	89.5 ± 7.7	90.2 ± 6.9
	BUP04^a^	91.8 ± 6.0	90.8 ± 3.9	94.0 ± 1.4
$\text{C}\overset{-}{\text{v}}{\text{O}}_{\text{2}}$ (mL dL^-1^)	BUP02	16.3 ± 2.2	14.3 ± 1.9*	14.5 ± 2.1*
	BUP04^a^	15.2 ± 1.4	13.8 ± 2.1*	14.4 ± 1.5*

PaO_2_ = arterial partial pressure of oxygen; CaO_2_ = arterial content of oxygen; DO_2_I = oxygen delivery index; VO_2_I = oxygen consumption index; O_2_ER = oxygen extraction ratio; $\text{P}\overset{-}{\text{v}}{\text{O}}_{\text{2}}$ = mixed venous partial pressure of oxygen; $\text{C}\overset{-}{\text{v}}{\text{O}}_{\text{2}}$ = mixed venous oxygen content; $\text{S}\overset{-}{\text{v}}{\text{O}}_{\text{2}}$ = mixed venous oxygen saturation; Hba = hemoglobin concentration in arterial blood.

^a^ = data shown for only 4 dogs;

* = significant difference between T0;. p < 0.05.

**Fig 1:**
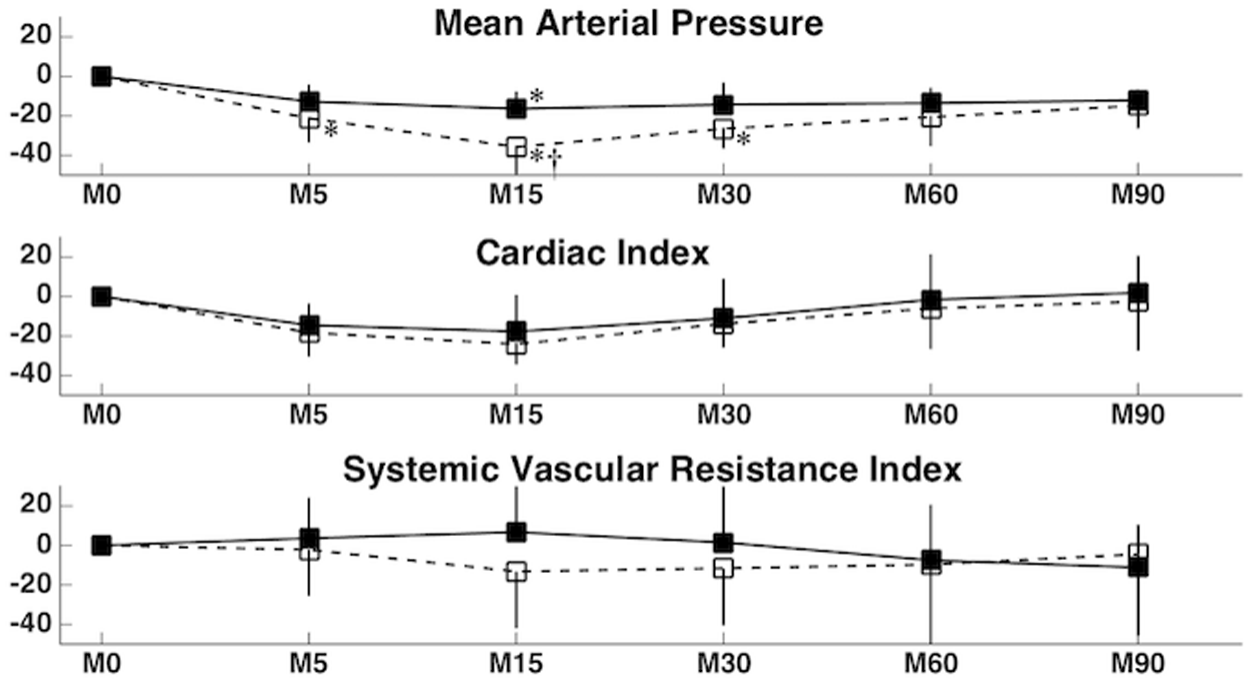
Percentage changes in mean arterial pressure (MAP) and its determinants, cardiac index and systemic vascular resistance index, after the administration of two volumes of epidural bupivacaine (0.25%) in six dogs anesthetized with 1.3 minimum alveolar concentration of isoflurane. * = Significant difference from T0; † = significant difference between BUP02 and BUP04. p < 0.05.

The percentage decreases in MAP, CI, and SVRI are presented in Fig 1. The only significant difference found was in MAP between the groups at T15 (p = 0.0019), and within each group at T15 for BUP02 (p = 0.0225) and T5, T15 and T30 for BUP04 (p = 0.0112). Mild hypotension (MAP = 59 mmHg) was recorded only in one dog with BUP02 at 15 minutes. In BUP04, one dog had moderate hypotension (MAP = 48 mmHg) at T5; 3 dogs presented with mild hypotension (MAP = 50, 54 and 59 mmHg) and 1 dog moderate hypotension (MAP = 49 mmHg) at T15; and one dog presented with mild hypotension (MAP = 53 mmHg) at T30 and T60.

Two dogs in BUP04 had MAP lower than 50 mmHg at T15 and were mechanically ventilated due to hypoventilation. The data from these two dogs were excluded from the statistical analysis. All animals from both groups had signs of residual motor block in the pelvic limbs, and two dogs from BUP04 had classical signs of Horner’s syndrome such as ptosis, enophthalmia, miosis and prominent third eyelid. All signs resolved spontaneously within 4 hours.

## Discussion

There were 5 main findings of the present study of dogs anesthetized with 1.3 MAC of isoflurane and bupivacaine 0.25% epidural administration of 0.2 or 0.4 mL kg^-1^: 1) A volume-dependent decrease in MAP; 2) occurrence of hypotension was higher in BUP04 than BUP02; 3) hypoventilation was observed during the initial 15 minutes with BUP04, which was associated with a decrease in V_T_, *f*_R_ and consequently in ${\dot{\text{V}}}_{\text{E}}$; 4) CaO_2_ decreased in both treatments mainly because of a decrease in hemoglobin concentration; and 5) the Horner’s syndrome observed in two dogs of BUP04 indicated that the administration of 0.4 mL kg^-1^ can reach high thoracic levels of blockade (T1—T2—T3).

There is a wide range of volumes recommended for the lumbosacral epidural injection of local anesthetics in dogs [1, 8, 15]. Higher volumes of local anesthetics, like the one used in BUP04, may be clinically applicable to procedures in the cranial abdomen and thorax as the volume of epidural injection is one of the main determinants of the cranial spread of epidural anesthesia [12, 15, 28, 29]. Nevertheless, the cardiovascular and respiratory effects were more significant with BUP04 and need to be taken into consideration for its rational clinical application.

Even though the authors were not able to find a direct comparison of the cardiovascular and respiratory effects of bupivacaine at different concentrations, the motor and sensory block was shorter and less intense with 0.25% than with 0.5 and 0.75% [14]. This suggests that the cardiovascular and respiratory effects of epidural bupivacaine reported in this study can be more pronounced and prolonged when using a concentration higher than 0.25%.

Even though epidural bupivacaine is commonly used in dogs in combination with inhalant anesthetics as a balanced anesthetic technique [1, 30], its cardiovascular and respiratory effects in these conditions have not been fully characterized, especially at 0.25%. Previous studies demonstrated the cardiovascular effects of the epidural administration of 0.5 and 0.75% bupivacaine in dogs [21, 22] but the conditions under these investigations were greatly different from the clinical scenario. Bradycardia and hypotension from the epidural administration of 0.5% bupivacaine have been previously reported in isoflurane-anesthetized dogs [3, 9]. However, a more detailed description of the cardiovascular effects of epidural bupivacaine in combination with inhalant anesthetics is warranted for a better understanding of the pathophysiology and treatment of cardiovascular depression.

The present study aimed to investigate the cardiovascular and respiratory effects of the epidural administration of bupivacaine in conditions that could provide better clinical application than in previous studies [12, 19–21]. However, the anesthetic maintenance of a constant FE´ISO of 1.3 MAC of isoflurane and the positioning the dog in sternal recumbency during the experiments are conditions dissimilar to the ones found in the clinical scenario. Consequently, these are limitations of this study and the direct application of the results of this study should be carefully considered.

The FE´ISO could likely be decreased after the administration of bupivacaine in clinical patients because the epidural administration of local anesthetics can decrease the MAC of inhalant anesthetics [2]. However, the cardiovascular and respiratory effects of epidural bupivacaine were better isolated and characterized with a constant FE´ISO. Due to the dose-dependent cardiovascular and respiratory depression of isoflurane [31], the observed hypoventilation and decrease in MAC could possibly be minimized if the isoflurane concentration had been decreased after both doses of epidural bupivacaine. 1.3 isoflurane MAC is associated with moderate levels of anesthesia [32] and was chosen in this study because when lower FE´ISO was used in the pilot study, the dogs presented peaks of MAP and PR associated with signs of light anesthesia.

The positioning of the dog in sternal recumbency during the experiments may not reproduce the recumbency used for most orthopedic procedures that would indicate an epidural administration of bupivacaine (i.e. lateral or dorsal recumbency). However, sternal recumbency is used for some soft tissue surgeries such as perineal herniorrhaphy. Sternal recumbency was chosen to perform the epidural administration of bupivacaine because it is very commonly used for this purpose in the clinical scenario [8]. Alternatively, the dog could have been placed in dorsal recumbency right after the epidural administration of bupivacaine but this maneuver was not performed for two main reasons: 1) possible dislodging of the PA catheter and other components of the instrumentation and measurements; and 2) the change in cardiovascular and respiratory function associated with this change in recumbency. Maintaining the dog in one position at all time points of data collection allowed us to isolate the cardiovascular and respiratory effects of the epidural treatments and the pathophysiology of the dose dependent effects of epidural bupivacaine in isoflurane-anesthetized dogs presented here are still applicable to the clinical settings. The cardiovascular depression from the epidural administration of bupivacaine observed in the present study is probably potentiated in dorsal recumbency which has been associated with worse cardiovascular and respiratory performance when compared to lateral or sternal recumbency [33, 34].

The epidural administration of approximately 0.25 mL kg^-1^ of 0.75% bupivacaine in awake dogs caused a decrease in MAP due to a decrease in CO with no effect on SVR [21]. Nevertheless, decreases in SVR and CO were responsible for the decrease in MAP when 0.5% bupivacaine (0.5–0.6 mL kg^-1^) was administered by the same route in dogs anesthetized with thiopental [20]. It is yet to be determined if the presence of a general anesthetic, a higher volume of bupivacaine used in anesthetized dogs, or a combination of both factors is responsible for the difference between the results of these two studies. Indeed, the addition of a general anesthetic can increase the odds of developing significant hypotension [22, 23], likely by the inhibition of increased release of vasopressin that seems to compensate for decreased SVR in the awake state. Similar to both previous studies, a decrease in MAP was also observed in the dogs of the present study after the epidural administration of bupivacaine 0.25%. The occurrence of moderate and mild hypotension was higher in BUP04 than in BUP02, where only one dog presented mild hypotension 15 minutes after the epidural treatment.

The two determinants of MAP (CI and SVRI) decreased significantly in the previous study with thiopental [21] but neither of these determinants decreased in the present study. However, based on a post-hoc power analysis, the exclusion of data from 2 dogs of BUP04 underpowered the study to identify statistical significances when differences between or within treatments were less than 30%. It is not certain whether or not statistical significance in CI and SVRI between and within treatments would have been observed if the two dogs of BUP04 had not been excluded. It is also possible that the effects of the epidural treatments on CI and SVRI are minimal but a little more pronounced in the dogs of BUP04 than in BUP02. In this case, the observation of a significant decrease in MAP, especially in the dogs of BUP04, was a result of the summation of small and statistically nonsignificant decreases in CI and SVRI. The interpretation of the cardiovascular results of this study has a very important clinical implication on the treatment of hypotension during isoflurane anesthesia in dogs. Because the decrease in MAP observed in both epidural volumes of bupivacaine 0.25% had no predominance of vasodilation over decreases in CI, the use of a positive inotrope to improve CI should be considered in combination or not with vasoconstrictors when hypotension is observed after the epidural administration of bupivacaine in isoflurane anesthetized dogs. The quantification of plasma catecholamine levels could have helped to elucidate the effects of epidural bupivacaine in the adrenal function and would have provided a better understanding of the cardiovascular effects of each treatment used.

The sympathetic blockade caused by the epidural administration of local anesthetics has been indirectly demonstrated by the suppression of sympathetic responses like the one from CO_2_ [11, 35] as well as by changes in the skin temperature [36]. Because the cranial spread of local anesthetics at the same concentration increases as the volume increases [16, 17, 27], the more pronounced hypotension observed with the higher volume of epidural bupivacaine seemed to be related to a higher sympathetic blockade. The results of this study support the contraindication of the epidural administration of bupivacaine in hypotensive and/or hypovolemic patients suggested by some authors [8] because the hypotensive effects observed in the present study could be more accentuated in these patients.

The more cranial spread of bupivacaine in BUP04 compared to BUP02 seemed to also be the reason why hypoventilation was observed only in the dogs of BUP04. The motor block caused by bupivacaine at the intercostal nerves was probably the main reason for the hypoventilation observed in BUP04. Even though the possibility of a partial block of the phrenic nerve at the level of C5 –C7 is low, it cannot be totally excluded as an additional cause of the more pronounced hypoventilation in the dogs of BUP04. The cervical and thoracic epidural injection of mepivacaine caused hypoventilation in humans by a significant decrease in V_T_ [18] as observed in the dogs of BUP04. The other potential cause of the hypoventilation observed with BUP04 can be associated with the decreased perfusion of the brainstem related to low MAP observed in the dogs that received the higher volume of bupivacaine [37]. The decreased or absent sympathetic response to CO_2_ in the presence of high thoracic and cervical levels of epidural local anesthetics can cause significant hypotension in association with hypoventilation [11, 33]. This was the reason why mechanical ventilation was initiated if hypotension occurred simultaneously to hypoventilation. Indeed, the MAP improved in two dogs of BUP04 from the initiation of positive pressure ventilation and did not require additional treatment similar to two dogs where a high volume of epidural bupivacaine was administered [11]. A significant decrease in lung volumes and capacities with a resultant decrease in arterial oxygen partial pressure and increase in the gradient between alveolar and arterial oxygen partial pressures (PaO_2_) has been reported after the cervical and thoracic epidural mepivacaine in humans [18]. Nevertheless, any change in PaO_2_ was detected after the epidural administration of either volume of bupivacaine.

In the present study, CaO_2_ decreased after the administration of both bupivacaine treatments as a result of a decrease in Hba concentration. Decrement in Hba and consequently decrement in CaO_2_ may be possible because of the high epidural bupivacaine blocking splanchnic sympathetic discharge (affecting regional resistance through loss of vasoconstrictive activity) causing reduction in portal venous flow and total hepatic blood flow [21]. However, no effect on splanchnic blood flow was observed in a study with upper thoracic epidural lidocaine in propofol-anesthetized dogs [38]. Different from the findings of this study, no significant difference in Hba was observed in a study with 0.2 mL kg^-1^ epidural 0.5% bupivacaine in conscious dogs [39]. The decrements in CaO_2_ and Hba could also be partially attributed to anesthesia since these parameters were also decreased in isoflurane anesthetized dogs that received an epidural injection of saline [40]. However, Hba did not decrease in dogs anesthetized with 1.3 isoflurane MAC within the timeframe of the present study [41]. The lack of a control group limits the ability to understand if the decrease of Hba was due to a temporal effect or caused by the treatments. Even though, the mild decrease in Hba and CaO_2_ observed in both epidural treatments was not enough to cause a statistical decrease in IDO_2_, an approximately 20% decrease was observed in both treatments. Likely, this decrease in DO_2_I was not clinically significant because no decrease in $\text{S}\overset{-}{\text{v}}{\text{O}}_{\text{2}}$ was observed.

Horner’s syndrome was observed in 2 dogs from BUP04 group and it can be related to the local anesthetic achieving cranial thoracic dermatomes (T1—T2—T3) [12, 36]. These clinical signs are related to the interruption of the ocular sympathetic innervation as the local anesthetic spreads through the spinal cord, including miosis, ptosis, enophthalmia and prolapse of the third eyelid [42]. The Horner’s syndrome observed in both dogs of this study resolved spontaneously within 4 hours after anesthetic recovery.

One of the major limitations of this study is the absence of bupivacaine plasma concentration measurement, which could improve the understanding of the influence of the systemic effects of bupivacaine on the results of the present study. The plasma concentration of bupivacaine achieved when a dose of bupivacaine approximately twice as high as the one used in BUP04 was used in the epidural space of dogs [20] was much lower than the one achieving significant cardiovascular depression [43]. Consequently, the contribution of the cardiovascular effects observed in the present study from the systemic uptake of bupivacaine is expected to be minimal. The results of this study should be applied with caution in dogs of different body weights and vertebral column morphology because the cranial spread of a local anesthetic solution does not obey a linear relationship with body weight as proposed by Otero et al. [27]. Lastly, the use of mechanical ventilation as opposed to spontaneous ventilation may modify the responses observed in the present study. The initiation of mechanical ventilation in two dogs that presented moderate hypotension provided an improvement of MAP, as observed when a higher volume of 0.25% bupivacaine was administered in a similar condition to the present study [11].

## Conclusion

In conclusion, the main cardiovascular effect of the epidural administration of 0.25% bupivacaine in dogs anesthetized with 1.3 MAC of isoflurane was a dose-dependent decrease in MAP, with a higher occurrence of hypotension when 0.4 mL kg^-1^ was used. A decrease in CaO_2_ due to a decrease in hemoglobin concentration was observed independently of the volume of epidural bupivacaine. Hypoventilation was only observed when 0.4 mL kg^-1^ of epidural bupivacaine was administered.

## Supporting information

S1 File Cardiovascular and respiratory effects of 0.2 mL/kg of epidural bupivacaine (0.25%) in six dogs anesthetized with 1.3 minimum alveolar concentration of isoflurane. T0 = before epidural administration. T5, T15, T30, T60 and T90 are 5, 15, 30, 60 and 90 minutes after the epidural treatment. SD = standard deviation, Q1 = first quartile, Q3 = third quartile.
(PDF)

S2 File Cardiovascular and respiratory effects of 0.4 mL/kg of epidural bupivacaine (0.25%) in six dogs anesthetized with 1.3 minimum alveolar concentration of isoflurane. T0 = before epidural administration. T5, T15, T30, T60 and T90 are 5, 15, 30, 60 and 90 minutes after the epidural treatment. The values in red were from animals that received mechanical ventilation and were excluded from the final statistical analysis. SD = standard deviation, Q1 = first quartile, Q3 = third quartile.
(PDF)

S3 File Cardiovascular and respiratory effects of 0.4 mL/kg of epidural bupivacaine (0.25%) in six dogs anesthetized with 1.3 minimum alveolar concentration of isoflurane. T0 = before epidural administration. T5, T15, T30, T60 and T90 are 5, 15, 30, 60 and 90 minutes after the epidural treatment. The values from the mechanically ventilated dogs were not reported. SD = standard deviation, Q1 = first quartile, Q3 = third quartile.
(PDF)
