# Supplementary material for: Cardiovascular and respiratory effects of lumbosacral epidural bupivacaine in isoflurane-anesthetized dogs: The effects of two volumes of 0.25% solution
Source: PLoS One. 2018 Apr 18;13(4):e0195867. doi: 10.1371/journal.pone.0195867 (PMC5906007; doi:10.1371/journal.pone.0195867)
Supplement: S1 File — T0 = before epidural administration. T5, T15, T30, T60 and T90 are 5, 15, 30, 60 and 90 minutes after the epidural treatment. SD = standard deviation, Q1 = first quartile, Q3 = third quartile. (PDF) [file pone.0195867.s001.pdf]

Cardiovascular and Respiratory effects of 0.2 mL/kg of epidural bupivacaine (0.25%) in six dogs anesthetized with 1.3 minimum alveolar concentration of isoflurane.

PULSE RATE (beats/min)

	Time Points	T0	T5	T15	T30	T60	T90
Dog	a	160	144	138	147	151	160
	b	138	117	113	114	115	118
	c	147	139	137	136	139	138
	d	136	136	121	122	125	127
	e	121	115	112	112	114	114
	f	134	138	147	155	159	143
	Median	137	137	129	129	132	133
	Q1	135	122	115	116	118	120
	Q3	145	139	138	144	148	142

Mean Arterial Pressure (mmHg)

		T0	T5	T15	T30	T60	T90
Dog	a	110	102	103	105	98	101
	b	80	66	59	60	66	70
	c	102	81	80	78	78	80
	d	88	88	82	89	83	79
	e	72	64	60	62	68	64
	f	100	80	79	80	82	91
	Mean	92.0	80.2	77.2	79.0	79.2	80.8
	SD	14.5	14.1	16.3	16.9	11.6	13.5

Central Venous Pressure (mmHg)

		T0	T5	T15	T30	T60	T90
Dog	a	3	1	1	1	1	1
	b	5	5	5	5	5	6
	c	3	3	5	3	4	3
	d	6	6	6	6	5	6
	e	6	6	6	5	6	7
	f	4	3	3	5	4	3
	Mean	4.5	4.0	4.3	4.2	4.2	4.3
	SD	1.4	2.0	2.0	1.8	1.7	2.3

Cardiac Index (L/min/m2)

	Time Points	T0	T5	T15	T30	T60	T90
Dog	a	7.71	7.59	7.36	7.44	9.23	9.35
	b	2.81	2.16	2.07	2.07	2.27	3.04
	c	3.55	3.25	3.10	3.47	3.40	3.99
	d	5.18	3.61	2.67	3.17	3.45	3.53
	e	3.59	3.22	2.99	3.11	3.60	3.59
	f	5.77	4.99	5.90	6.81	7.34	5.86
	Mean	4.77	4.14	4.01	4.34	4.88	4.89
	SD	1.82	1.92	2.11	2.22	2.75	2.39

Stroke Index (mL/kg)							
Time Points		T0	T5	T15	T30	T60	T90
Dog	a	1.83	2.00	2.02	1.92	2.32	2.21
	b	0.72	0.65	0.64	0.64	0.69	0.91
	c	1.01	0.98	0.95	1.06	1.02	1.21
	d	1.45	1.01	0.84	0.99	1.05	1.06
	e	1.12	1.06	1.01	1.05	1.20	1.19
	f	1.72	1.45	1.61	1.76	1.85	1.64
	Mean	1.31	1.19	1.18	1.24	1.35	1.37
	SD	0.43	0.47	0.52	0.49	0.61	0.48

Systemic Vascular Resistance Index (dynes s/cm5/m2)							
		T0	T5	T15	T30	T60	T90
Dog	a	1110	1064	1109	1118	841	856
	b	2132	2259	2090	2129	2154	1686
	c	2232	1919	1934	1731	1741	1542
	d	1266	1817	2280	2093	1811	1655
	e	1472	1441	1445	1465	1378	1272
	f	1332	1233	1030	881	850	1202
	Mean	1,591	1,622	1,648	1,570	1,463	1,369
	SD	474	453	528	510	538	321

Left Ventricle Stroke work index (cJ/kg)							
Time Points		T0	T5	T15	T30	T60	T90
Dog	a	2.48	2.55	2.67	2.58	2.84	2.80
	b	0.67	0.51	0.46	0.46	0.54	0.75
	c	1.28	1.00	0.93	1.03	0.99	1.20
	d	1.54	1.07	0.82	1.06	1.07	0.99
	e	0.90	0.75	0.65	0.73	0.91	0.84
	f	2.04	1.36	1.47	1.65	1.76	1.76
	Mean	1.49	1.21	1.16	1.25	1.35	1.39
	SD	0.69	0.72	0.81	0.76	0.83	0.78

Mean Pulmonary Artery Pressure (mmHg)							
		T0	T5	T15	T30	T60	T90
Dog	a	21.0	17.0	15.0	15.0	16.0	18.0
	b	18.0	15.0	14.0	13.0	14.0	16.0
	c	15.0	14.0	15.0	14.0	15.0	14.0
	d	18.0	17.0	15.0	15.0	16.0	15.0
	e	20.0	20.0	19.0	20.0	20.0	21.0
	f	19.0	17.0	17.0	19.0	19.0	17.0
	Mean	18.5	16.7	15.8	16.0	16.7	16.8
	SD	2.1	2.1	1.8	2.8	2.3	2.5

Pulmonary Vascular Resistance Index (dynes s/cm5/m2)

		T0	T5	T15	T30	T60	T90
Dog	a	114	95	98	97	69	86
	b	199	259	271	232	177	184
	c	135	197	180	162	188	140
	d	124	155	150	126	186	113
	e	156	199	161	231	178	201
	f	83	96	68	94	76	68
	Mean	135	167	155	157	146	132
	SD	39	64	71	63	57	53

Right Ventricle Stroke work index (cJ/kg)

	Time Points	T0	T5	T15	T30	T60	T90
Dog	a	0.45	0.43	0.38	0.37	0.47	0.51
	b	0.13	0.09	0.08	0.07	0.08	0.12
	c	0.16	0.15	0.13	0.16	0.15	0.18
	d	0.24	0.15	0.10	0.12	0.16	0.13
	e	0.21	0.20	0.18	0.21	0.23	0.23
	f	0.35	0.28	0.31	0.34	0.38	0.31
	Mean	0.26	0.22	0.20	0.21	0.25	0.25
	SD	0.12	0.12	0.12	0.12	0.15	0.15

RESPIRATORY RATE (BREATHS/MIN)

	Time Points	T0	T5	T15	T30	T60	T90
Dog	a	16	11	14	17	17	17
	b	18	12	9	12	12	12
	c	16	13	12	12	11	9
	d	11	11	13	16	13	15
	e	14	14	10	11	11	10
	f	15	15	15	13	18	15
	Mean	15	13	12	14	14	13
	SD	2	2	2	2	3	3

TIDAL VOLUME (ML/KG)

	Time Points	T0	T5	T15	T30	T60	T90
Dog	a	21	19	18	19	18	17
	b	10	10	11	9	12	13
	c	13	12	14	14	15	15
	d	11	12	13	13	13	14
	e	12	12	13	14	16	15
	f	13	14	14	14	12	16
	Mean	13.3	13.1	13.8	13.7	14.6	15.1
	SD	3.9	3.1	2.4	3.1	2.5	1.3

MINUTE VENTILATION (mL/kg/min)

	Time Points	T0	T5	T15	T30	T60	T90
Dog	a	377	225	162	228	218	199
	b	160	130	126	111	133	116
	c	143	127	179	217	200	231
	d	160	166	130	140	148	144
	e	179	187	202	178	295	232
	f	192	180	168	191	166	205
	Mean	202	169	161	178	193	188
	SD	87	37	29	45	59	48

Arterial partial pressure of CO2 (mmHg)

	Time Points	T0	T5	T15	T30	T60	T90
Dog	a	50.2		49.8		49.4	
	b	59		61.2		61.8	
	c	52.2		54.5		55.7	
	d	52.3		56.1		53.7	
	e	58.9		58.9		59.3	
	f	51.2		45		44.2	
	Mean	54.0		54.3		54.0	
	SD	3.9		6.0		6.5	

Arterial Bicarbonate (mmol/L)

	Time Points	T0	T5	T15	T30	T60	T90
Dog	a	25.7		24.2		24.6	
	b	25.1		25.5		26	
	c	25.4		26.5		26.9	
	d	25		25.2		25.4	
	e	25.1		23.7		25.1	
	f	22.5		22		22.8	
	MEDIAN	25.1		24.7		25.3	
	Q1	25.0		23.8		24.7	
	Q2	25.3		25.4		25.9	

arterial pH

	Time Points	T0	T5	T15	T30	T60	T90
Dog	a	7.32		7.299		7.321	
	b	7.23		7.23		7.23	
	c	7.298		7.3		7.296	
	d	7.292		7.264		7.29	
	e	7.181		7.22		7.241	
	f	7.255		7.302		7.325	
	MEDIAN	7.274		7.282		7.293	
	Q1	7.236		7.239		7.253	
	Q2	7.297		7.300		7.315	

Base excess (mmol/L)

	Time Points	T0	T5	T15	T30	T60	T90
Dog	a	0		-2		-1	
	b	-2		-2		-1	
	c	-1		0		1	
	d	-1		-2		-1	
	e	-3		-4		-2	
	f	-4		-4		-3	
	Mean	-1.8		-2.3		-1.2	
	SD	1.5		1.5		1.3	

Arterial partial pressure of O2 (mmHg)

	Time Points	T0	T15	T60
Dog	a	492	540	572
	b	574	527	541
	c	533	572	533
	d	525	565	570
	e	495	502	472
	f	540	528	561
	Média	527	539	542
	DP	31	26	37

Arterial Hemoglobin (g/dL)

	Time Points	T0	T15	T60
Dog	a	13.9	12.2	12.9
	b	12.9	11.6	11.2
	c	12.2	11.3	10.9
	d	11.6	11.2	11.2
	e	11.2	10.2	10.2
	f	11.9	10.9	11.6
	Média	12.3	11.2	11.3
	DP	1.0	0.7	0.9

Arterial O2 content (mL/dL)

	Time Points	T0	T15	T60
Dog	a	20.01	18.22	18.73
	b	19.71	17.76	17.25
	c	18.19	17.34	16.80
	d	17.75	17.32	17.34
	e	17.10	14.34	15.22
	f	17.24	16.23	17.86
	Mean	18.3	16.9	17.2
	SD	1.2	1.4	1.2

O2 delivery index (mL/min/m2)

	Time Points	T0	T15	T60
Dog	a	1544	1341	1729
	b	555	367	391
	c	646	601	671
	d	920	549	612
	e	613	446	546
	f	994	1105	1046
	Média	879	735	832
	DP	371	394	490

Oxygen consumption index (mL/min/m2)

	Time Points	T0	T15	T60
Dog	a	54.66	63.71	112.86
	b	52.66	59.92	97.26
	c	89.73	97.68	104.74
	d	119.85	108.04	115.09
	e	154.31	82.93	116.94
	f	39.12	81.76	86.61
	Mean	85	82	106
	SD	45	19	12

O2 extraction ratio

	Time Points	T0	T15	T60
Dog	a	0.04	0.05	0.07
	b	0.09	0.16	0.25
	c	0.14	0.16	0.16
	d	0.13	0.20	0.19
	e	0.25	0.19	0.21
	f	0.04	0.07	0.08
	Mean	0.12	0.14	0.16
	SD	0.08	0.06	0.07

Mixed venous partial pressure of O2 (mmHg)

	Time Points	T0	T15	T60
Dog	a	119	181	95
	b	86	58	60
	c	75	74	78
	d	91	69	70
	e	61	63	63
	f	114	108	136
	Median	89	72	74
	Q1	78	65	65
	Q3	108	100	91

Mixed venous O2 Saturation (%)

	Time Points	T0	T15	T60
Dog	a	98	99	96
	b	98	81	82
	c	91	91	92
	d	94	88	89
	e	81	81	83
	f	98	97	99
	Mean	93.3	89.5	90.2
	SD	6.7	7.7	6.9

Mixed Venous O2 content (mL/dL)

	Time Points	T0	T15	T60
Dog	a	19.30	17.35	17.51
	b	17.84	13.24	12.95
	c	15.66	14.52	14.18
	d	15.44	13.91	14.07
	e	12.80	11.68	11.96
	f	16.56	15.03	16.38
	Mean	16.3	14.3	14.5
	SD	2.2	1.9	2.1
