# Supplementary material for: Cardiovascular and respiratory effects of lumbosacral epidural bupivacaine in isoflurane-anesthetized dogs: The effects of two volumes of 0.25% solution
Source: PLoS One. 2018 Apr 18;13(4):e0195867. doi: 10.1371/journal.pone.0195867 (PMC5906007; doi:10.1371/journal.pone.0195867)
Supplement: S2 File — T0 = before epidural administration. T5, T15, T30, T60 and T90 are 5, 15, 30, 60 and 90 minutes after the epidural treatment. The values in red were from animals that received mechanical ventilation and were excluded from the final statistical analysis. SD = standard deviation, Q1 = first quartile, Q3 = third quartile. (PDF) [file pone.0195867.s002.pdf]

Cardiovascular and Respiratory effects of 0.4 mL/kg of epidural bupivacaine (0.25%) in six dogs anesthetized with 1.3 minimum alveolar concentration of isoflurane.

The values in Red were from animals that received mechanical ventilation and were excluded from the final statistical analysis

PULSE RATE (beats/min)

Time Points		T0	T5	T15	T30	T60	T90
Dog	a	141	120	109	114	123	128
	b	125	117	109	108	108	108
	c	134	121	118	122	123	124
	d	121	108	108	111	113	113
	e	111	110	97	95	97	103
	f	125	116	121	121	122	124
	Median	125	117	109	113	118	119
	Q1	122	112	108	109	109	109
	Q3	132	119	116	119	123	124

Mean Arterial Pressure (mmHg)

	Time Points	T0	T5	T15	T30	T60	T90
Dog	a	98	70	50	68	82	93
	b	73	70	59	64	71	69
	c	94	61	54	64	62	72
	d	66	48	61	60	64	70
	e	83	68	49	53	53	60
	f	103	82	70	71	73	77
	Mean	86	66.5	57.2	63.3	67.5	73.5
	SD	15	11.3	7.9	6.3	10.1	11.0

Central Venous Pressure (mmHg)

	Time Points	T0	T5	T15	T30	T60	T90
Dog	a	2	3	3	2	3	1
	b	6	5	2	3	3	3
	c	1	1	1	1	3	1
	d	4	5	4	4	8	8
	e	4	5	5	6	4	5
	f	5	4	4	5	4	5
	Mean	3.7	3.8	3.2	3.5	4.2	3.8
	SD	1.9	1.6	1.5	1.9	1.9	2.7

Cardiac Index (L/min/m2)

	Time Points	T0	T5	T15	T30	T60	T90
Dog	a	4.83	3.40	3.36	4.33	5.64	5.85
	b	6.66	4.95	4.38	4.60	4.46	4.17
	c	3.91	3.83	3.25	3.68	3.71	4.14
	d	4.02	3.46	3.68	3.80	4.03	4.54
	e	2.45	2.19	2.12	2.19	2.14	2.53
	f	5.15	3.93	4.39	4.72	4.99	5.15
	Mean	4.50	3.63	3.53	3.89	4.16	4.40
	SD	1.41	0.90	0.84	0.93	1.21	1.12

Stroke Index (mL/kg)

	Time Points	T0	T5	T15	T30	T60	T90
Dog	a	1.30	1.07	1.17	1.44	1.74	1.73
	b	1.88	0.75	0.71	0.76	0.73	0.68
	c	1.22	1.32	1.15	1.26	1.26	1.40
	d	1.27	1.22	1.30	1.30	1.36	1.53
	e	0.84	0.76	0.83	0.87	0.84	0.93
	f	1.80	1.48	1.58	1.70	1.79	1.81
	Mean	1.38	1.10	1.12	1.22	1.29	1.35
	SD	0.39	0.30	0.32	0.35	0.44	0.45

Systemic Vascular Resistance Index (dynes s/cm5/m2)

	Time Points	T0	T5	T15	T30	T60	T90
Dog	a	1590	1578	1120	1219	1120	1259
	b	805	1050	1041	1061	1220	1267
	c	1902	1254	1304	1370	1271	1371
	d	1234	994	1240	1179	1111	1093
	e	2580	2299	1657	1715	1833	1739
	f	1524	1586	1203	1118	1105	1119
	Mean	1606	1460	1261	1277	1277	1308
	SD	604	482	215	239	281	235

Left Ventricle Stroke work index (cJ/kg)

	Time Points	T0	T5	T15	T30	T60	T90
Dog	a	1.55	0.90	0.67	1.19	1.75	2.05
	b	1.63	0.66	0.51	0.61	0.65	0.59
	c	1.43	0.92	0.74	0.99	0.96	1.22
	d	1.02	0.70	0.94	0.92	1.04	1.29
	e	0.81	0.61	0.45	0.55	0.52	0.66
	f	2.15	1.41	1.25	1.34	1.44	1.58
	Mean	1.43	0.87	0.76	0.93	1.06	1.23
	SD	0.48	0.30	0.30	0.32	0.47	0.55

Mean Pulmonary Artery Pressure (mmHg)

	Time Points	T0	T5	T15	T30	T60	T90
Dog	a	17	15	13	15	15	16
	b	21	15	15	14	13	13
	c	15	14	12	12	13	15
	d	15	14	14	16	15	17
	e	16	13	12	12	12	14
	f	18	16	16	16	16	16
	Mean	17.0	14.5	13.7	14.2	14.0	15.2
	SD	2.3	1.0	1.6	1.8	1.5	1.5

Pulmonary Artery Occlusion Pressure (mmHg)

	Time Points	T0	T5	T15	T30	T60	T90
Dog	a	10	8	8	7	8	6
	b	9	5	6	5	6	6
	c	8	10	7	6	6	8
	d	7	6	8	8	8	8
	e	12	9	9	7	7	8
	f	15	12	12	13	14	13
	Median	9.5	8.5	8.0	7.0	7.5	8.0
	Q1	8.3	6.5	7.3	6.3	6.3	6.5
	Q3	11.5	9.8	8.8	7.8	8.0	8.0

Pulmonary Vascular Resistance Index (dynes s/cm5/m2)

	Time Points	T0	T5	T15	T30	T60	T90
Dog	a	116	165	119	148	99	137
	b	144	162	164	156	126	134
	c	143	84	123	130	151	135
	d	159	185	131	168	139	159
	e	131	146	113	182	187	190
	f	47	81	73	51	32	47
	Mean	123	137	121	139	122	134
	SD	40	44	30	47	53	48

Right Ventricle Stroke work index (cJ/kg)

	Time Points	T0	T5	T15	T30	T60	T90
Dog	a	0.26	0.18	0.16	0.25	0.28	0.35
	b	0.38	0.10	0.13	0.11	0.10	0.09
	c	0.23	0.23	0.17	0.19	0.17	0.27
	d	0.19	0.15	0.18	0.21	0.13	0.19
	e	0.14	0.08	0.08	0.07	0.09	0.11
	f	0.32	0.24	0.26	0.26	0.29	0.27
	Mean	0.25	0.16	0.16	0.18	0.18	0.21
	SD	0.09	0.07	0.06	0.08	0.09	0.10

RESPIRATORY RATE (BREATHS/MIN)

	Time Points	T0	T5	T15	T30	T60	T90
Dog	a	19	9	9	12	17	19
	b	12	10	9	12	13	13
	c	23	9	10	13	14	12
	d	20	24	22	22	22	22
	e	11	14	19	18	19	19
	f	17	8	13	12	13	18
	Mean	17	12	14	15	16	17
	SD	5	6	6	4	4	4

TIDAL VOLUME (ML/KG)

	Time Points	T0	T5	T15	T30	T60	T90
	a	16	13	10	14	16	19
	b	8	7	6	9	10	12
Dog	c	14	12	11	13	16	16
	d	12	11	11	11	12	12
	e	12	9	12	12	11	11
	f	14	14	11	10	13	13
	Mean	12.8	10.8	10.1	11.4	12.8	13.8
	SD	2.9	2.7	2.0	1.7	2.4	3.0

MINUTE VENTILATION (mL/kg/min)

	Time Points	T0	T5	T15	T30	T60	T90
Dog	a	310	116	89	162	267	360
	b	95	67	56	111	131	150
	c	330	104	114	171	220	191
	d	245	263	239	248	254	274
	e	130	121	220	210	216	213
	f	242	112	141	116	163	232
	Mean	225	131	143	170	209	237
	SD	95	67	73	53	53	73

Arterial partial pressure of CO2 (mmHg)

	Time Points	T0	T5	T15	T30	T60	T90
	a	41		73		56.8	
	b	52.8		67.5		55.5	
	c	43		56.2		53.1	
Dog	d	48.9		48.5		51.5	
	e	53.6		46.0		47.4	
	f	48.4		53.7		55.1	
	Mean	48.0		57.5		53.2	
	SD	5.1		10.7		3.4	

Arterial Bicarbonate (mmol/L)

	Time Points	T0	T5	T15	T30	T60	T90
	a	20.7		19.9		23	
	b	23.2		20.0		22.2	
	c	23.6		24.0		26.5	
Dog	d	19.7		26.2		26.5	
	e	23.1		22.7		23	
	f	24.4		24.7		27.6	
	MEDIAN	23.2		23.4		24.8	
	Q1	21.3		20.7		23.0	
	Q2	23.5		24.5		26.5	

arterial pH

	Time Points	T0	T5	T15	T30	T60	T90
	a	7.31		7.10		7.28	
	b	7.25		7.13		7.22	
	c	7.35		7.34		7.3	
Dog	d	7.22		7.23		7.26	
	e	7.25		7.25		7.28	
	f	7.316		7.275		7.312	
	MEDIAN	7.280		7.240		7.280	
	Q1	7.250		7.155		7.265	
	Q2	7.315		7.269		7.295	

Base Excess (mmol/L)

	Time Points	T0	T5	T15	T30	T60	T90
Dog	a	-5		-2		0	
	b	-4		-6		-5	
	c	-2		-3		0	
	d	-3		-7		-4	
	e	-4.00		-4		-2	
	f	-2		-2		2	
	Mean	-3.3		-4.0		-1.5	
	SD	1.2		2.1		2.7	

Arterial partial pressure of O2 (mmHg)

	Time Points	T0	T15	T60
Dog	a	419	558	565
	b	482	504	474
	c	593	521	499
	d	546	396	579
	e	590	537	510
	f	587	541	585
	Média	536	510	535
	DP	71	59	47

Hemoglobin arterial (g/dL)

	Time Points	T0	T15	T60
Dog	a	12.2	10.9	11.6
	b	12.2	12.6	11.9
	c	11.2	9.2	9.5
	d	11.9	10.2	10.2
	e	11.2	9.5	8.8
	f	11.2	10.2	10.2
	Média	11.7	10.4	10.4
	DP	0.5	1.2	1.2

Arterial O2 concentration (mL/dL)

	Time Points	T0	T15	T60
Dog	a	17.42	16.32	18.29
	b	18.04	19.08	18.01
	c	16.99	14.40	15.31
	d	18.65	14.02	15.97
	e	17.40	13.06	12.98
	f	17.39	15.86	16.41
	Mean	17.6	15.5	16.2
	SD	0.6	2.1	1.9

O2 delivery index (mL/min/m2)

	Time Points	T0	T15	T60
Dog	a	842	548	1032
	b	1200	835	803
	c	664	468	568
	d	750	516	644
	e	426	278	277
	f	895	696	819
	Média	796.2	556.7	690.9
	DP	257.5	192.1	258.4

Oxygen consumption index (mL/min/m2)

	Time Points	T0	T15	T60
Dog	a	67.37	97.90	171.36
	b	66.83	109.20	97.23
	c	108.11	95.14	103.89
	d	163.39	64.38	137.86
	e	101.02	52.53	83.47
	f	74.37	92.48	124.86
	Mean	96.8	85.3	119.8
	SD	37.0	21.9	31.9

O2 extraction ratio

	Time Points	T0	T15	T60
Dog	a	0.08	0.18	0.17
	b	0.06	0.13	0.12
	c	0.16	0.20	0.18
	d	0.22	0.12	0.21
	e	0.24	0.19	0.30
	f	0.08	0.13	0.15
	Mean	0.14	0.16	0.19
	SD	0.08	0.03	0.06

Mixed venous partial pressure of O2 (mmHg)

	Time Points	T0	T15	T60
Dog	a	83	73	84
	b	133	95	91
	c	69	72	74
	d	63	69	71
	e	63	51	47
	f	171	90	96
	Median	76	73	79
	Q1	65	70	72
	Q3	121	86	89

Mixed venous O2 Saturation (%)

	Time Points	T0	T15	T60
Dog	a	93	87	93
	b	98	93	94
	c	90	88	93
	d	87	85	87
	e	84	79	73
	f	99	95	96
	Mean	91.8	87.8	89.3
	SD	6.0	5.7	8.5

Mixed Venous O2 content (mL/dL)

	Time Points	T0	T15	T60
Dog	a	16.03	13.41	15.26
	b	17.03	16.58	15.83
	c	14.23	11.48	12.51
	d	14.59	12.27	12.55
	e	13.27	10.59	9.08
	f	15.94	13.75	13.91
	Mean	15.2	13.0	13.2
	SD	1.4	2.1	2.4
