# Supplementary material for: Cardiovascular and respiratory effects of lumbosacral epidural bupivacaine in isoflurane-anesthetized dogs: The effects of two volumes of 0.25% solution
Source: PLoS One. 2018 Apr 18;13(4):e0195867. doi: 10.1371/journal.pone.0195867 (PMC5906007; doi:10.1371/journal.pone.0195867)
Supplement: S3 File — T0 = before epidural administration. T5, T15, T30, T60 and T90 are 5, 15, 30, 60 and 90 minutes after the epidural treatment. The values from the mechanically ventilated dogs were not reported. SD = standard deviation, Q1 = first quartile, Q3 = third quartile. (PDF) [file pone.0195867.s003.pdf]

Cardiovascular and Respiratory effects of 0.4 mL/kg of epidural bupivacaine (0.25%) in six dogs anesthetized with 1.3 minimum alveolar concentration of isoflurane

The values from the animals that received mechanical ventilation were excluded from the tables below

RESULTS EXCLUDING THE VENTILATED DOGS

PULSE RATE (beats/min)

Time Points		T0	T5	T15	T30	T60	T90
Dog	a	141	120	109	114	123	128
	b	125	117	109	108	108	108
	c	134	121	118	122	123	124
	d	121					
	e	111	110				
	f	125	116	121	121	122	124
	Median	125	117	114	118	123	124
	Q1	122	116	109	113	119	120
	Q3	132	120	119	121	123	125

Mean Arterial Pressure (mmHg)

	Time Points	T0	T5	T15	T30	T60	T90
Dog	a	98	70	50	68	82	93
	b	73	70	59	64	71	69
	c	94	61	54	64	62	72
	d	66					
	e	83	68				
	f	103	82	70	71	73	77
	Mean	86.2	70.2	58.3	66.8	72.0	77.8
	SD	14.7	7.6	8.7	3.4	8.2	10.7

Central Venous Pressure (mmHg)

	Time Points	T0	T5	T15	T30	T60	T90
Dog	a	2	3	3	2	3	1
	b	6	5	2	3	3	3
	c	1	1	1	1	3	1
	d	4					
	e	4	5				
	f	5	4	4	5	4	5
	Mean	3.7	3.6	2.5	2.8	3.3	2.5
	SD	1.9	1.7	1.3	1.7	0.5	1.9

Cardiac Index (L/min/m2)

	Time Points	T0	T5	T15	T30	T60	T90
Dog	a	4.83	3.40	3.36	4.33	5.64	5.85
	b	6.66	4.95	4.38	4.60	4.46	4.17
	c	3.91	3.83	3.25	3.68	3.71	4.14
	d	4.02					
	e	2.45	2.19				
	f	5.15	3.93	4.39	4.72	4.99	5.15
	Mean	4.50	3.66	3.84	4.33	4.70	4.83
	SD	1.41	1.00	0.63	0.47	0.82	0.82

Stroke Index (mL/kg)

	Time Points	T0	T5	T15	T30	T60	T90
Dog	a	1.30	1.07	1.17	1.44	1.74	1.73
	b	1.88	0.75	0.71	0.76	0.73	0.68
	c	1.22	1.32	1.15	1.26	1.26	1.40
	d	1.27					
	e	0.84	0.76				
	f	1.80	1.48	1.58	1.70	1.79	1.81
	Mean	1.38	1.08	1.15	1.29	1.38	1.41
	SD	0.39	0.33	0.36	0.40	0.49	0.51

Systemic Vascular Resistance Index (dynes s/cm5/m2)

	Time Points	T0	T5	T15	T30	T60	T90
	a	1590	1578	1120	1219	1120	1259
	b	805	1050	1041	1061	1220	1267
	c	1902	1254	1304	1370	1271	1371
	d	1234					
	e	2580	2299				
	f	1524	1586	1203	1118	1105	1119
	Mean	1606	1553	1167	1192	1179	1254
	SD	604	475	113	135	80	103

Left Ventricle Stroke work index (cJ/kg)

	Time Points	T0	T5	T15	T30	T60	T90
Dog	a	1.55	0.90	0.67	1.19	1.75	2.05
	b	1.63	0.66	0.51	0.61	0.65	0.59
	c	1.43	0.92	0.74	0.99	0.96	1.22
	d	1.02					
	e	0.81	0.61				
	f	2.15	1.41	1.25	1.34	1.44	1.58
	Mean	1.43	0.90	0.79	1.03	1.20	1.36
	SD	0.48	0.32	0.32	0.32	0.49	0.62

Mean Pulmonary Artery Pressure (mmHg)

	Time Points	T0	T5	T15	T30	T60	T90
Dog	a	17	15	13	15	15	16
	b	21	15	15	14	13	13
	c	15	14	12	12	13	15
	d	15					
	e	16	13				
	f	18	16	16	16	16	16
	Mean	17.0	14.6	14.0	14.3	14.3	15.0
	SD	2.3	1.1	1.8	1.7	1.5	1.4

Pulmonary Artery Occlusion Pressure (mmHg)

	Time Points	T0	T5	T15	T30	T60	T90
Dog	a	10	8	8	7	8	6
	b	9	5	6	5	6	6
	c	8	10	7	6	6	8
	d	7					
	e	12	9				
	f	15	12	12	13	14	13
	Median	9.5	9.0	7.5	6.5	7.0	7.0
	Q1	8.3	8.0	6.8	5.8	6.0	6.0
	Q3	11.5	10.0	9.0	8.5	9.5	9.3

Pulmonary Vascular Resistance Index (dynes s/cm5/m2)

	Time Points	T0	T5	T15	T30	T60	T90
Dog	a	116	165	119	148	99	137
	b	144	162	164	156	126	134
	c	143	84	123	130	151	135
	d	159					
	e	131	146				
	f	47	81	73	51	32	47
	Mean	123	127	120	121	102	113
	SD	40	42	37	48	51	44

Right Ventricle Stroke work index (cJ/kg)

	Time Points	T0	T5	T15	T30	T60	T90
Dog	a	0.26	0.18	0.16	0.25	0.28	0.35
	b	0.38	0.10	0.13	0.11	0.10	0.09
	c	0.23	0.23	0.17	0.19	0.17	0.27
	d	0.19					
	e	0.14	0.08				
	f	0.32	0.24	0.26	0.26	0.29	0.27
	Mean	0.25	0.17	0.18	0.20	0.21	0.25
	SD	0.09	0.07	0.06	0.07	0.09	0.11

RESPIRATORY RATE (BREATHS/MIN)

	Time Points	T0	T5	T15	T30	T60	T90
Dog	a	19	9	9	12	17	19
	b	12	10	9	12	13	13
	c	23	9	10	13	14	12
	d	20					
	e	11	14				
	f	17	8	13	12	13	18
	Mean	17	10	10	12	14	16
	SD	5	2	2	1	2	4

TIDAL VOLUME (ML/KG)

	Time Points	T0	T5	T15	T30	T60	T90
Dog	a	16	13	10	14	16	19
	b	8	7	6	9	10	12
	c	14	12	11	13	16	16
	d	12					
	e	12	9				
	f	14	14	11	10	13	13
	Mean	12.8	10.8	9.6	11.4	13.5	14.8
	SD	2.9	3.0	2.3	2.3	2.7	3.3

MINUTE VENTILATION (mL/kg/min)

	Time Points	T0	T5	T15	T30	T60	T90
Dog	a	310	116	89	162	267	360
	b	95	67	56	111	131	150
	c	330	104	114	171	220	191
	d	245					
	e	130	121				
	f	242	112	141	116	163	232
	Mean	225	104	100	140	195	233
	SD	95	21	36	31	60	91

Arterial partial pressure of CO2 (mmHg)

	Time Points	T0	T5	T15	T30	T60	T90
Dog	a	41		73		56.8	
	b	52.8		67.5		55.5	
	c	43		56.2		53.1	
	d	48.9					
	e	53.6					
	f	48.4		53.7		55.1	
	Mean	48.0		62.6		55.1	
	SD	5.1		9.2		1.5	

Arterial Bicarbonate (mmol/L)

	Time Points	T0	T5	T15	T30	T60	T90
Dog	a	20.7		19.9		23	
	b	23.2		20.0		22.2	
	c	23.6		24.0		26.5	
	d	19.7					
	e	23.1					
	f	24.4		24.7		27.6	
	MEDIAN	23.2		22.0		24.8	
	Q1	21.3		20.0		22.8	
	Q2	23.5		24.2		26.8	

arterial pH

	Time Points	T0	T5	T15	T30	T60	T90
Dog	a	7.31		7.10		7.28	
	b	7.25		7.13		7.22	
	c	7.35		7.34		7.3	
	d	7.22					
	e	7.25					
	f	7.316		7.275		7.312	
	MEDIAN	7.280		7.203		7.290	
	Q1	7.250		7.123		7.265	
	Q2	7.315		7.291		7.303	

Base Excess (mmol/L)

	Time Points	T0	T5	T15	T30	T60	T90
Dog	a	-5		-2		0	
	b	-4		-6		-5	
	c	-2		-3		0	
	d	-3					
	e	-4.00					
	f	-2		-2		2	
	Mean	-3.3		-3.3		-0.8	
	SD	1.2		1.9		3.0	

Arterial partial pressure of O2 (mmHg)

	Time Points	T0	T15	T60
Dog	a	419	558	565
	b	482	504	474
	c	593	521	499
	d	546		
	e	590		
	f	587	541	585
	Média	536	531	531
	DP	71	24	53

Hemoglobin arterial (g/dL)

	Time Points	T0	T15	T60
Dog	a	12.2	10.9	11.6
	b	12.2	12.6	11.9
	c	11.2	9.2	9.5
	d	11.9		
	e	11.2		
	f	11.2	10.2	10.2
	Média	11.7	10.7	10.8
	DP	0.5	1.4	1.1

Arterial O2 concentration (mL/dL)

	Time Points	T0	T15	T60
Dog	a	17.42	16.32	18.29
	b	18.04	19.08	18.01
	c	16.99	14.40	15.31
	d	18.65		
	e	17.40		
	f	17.39	15.86	16.41
	Mean	17.6	16.4	17.0
	SD	0.6	2.0	1.4

O2 delivery index (mL/min/m2)

	Time Points	T0	T15	T60
Dog	a	842	548	1032
	b	1200	835	803
	c	664	468	568
	d	750		
	e	426		
	f	895	696	819
	Média	796	637	806
	DP	258	162	190

Oxygen consumption index (mL/min/m2)

	Time Points	T0	T15	T60
Dog	a	67.37	97.90	171.36
	b	66.83	109.20	97.23
	c	108.11	95.14	103.89
	d	163.39		
	e	101.02		
	f	74.37	92.48	124.86
	Mean	97	99	124
	SD	37	7	33

O2 extraction ratio

	Time Points	T0	T15	T60
Dog	a	0.08	0.18	0.17
	b	0.06	0.13	0.12
	c	0.16	0.20	0.18
	d	0.22		
	e	0.24		
	f	0.08	0.13	0.15
	Mean	0.14	0.16	0.16
	SD	0.08	0.04	0.03

Mixed venous partial pressure of O2 (mmHg)

	Time Points	T0	T15	T60
Dog	a	83	73	84
	b	133	95	91
	c	69	72	74
	d	63		
	e	63		
	f	171	90	96
	Median	76	82	88
	Q1	65	73	82
	Q3	121	91	92

Mixed venous O2 Saturation (%)

	Time Points	T0	T15	T60
Dog	a	93	87	93
	b	98	93	94
	c	90	88	93
	d	87		
	e	84		
	f	99	95	96
	Mean	91.8	90.8	94.0
	SD	6.0	3.9	1.4

Mixed Venous O2 content (mL/dL)

	Time Points	T0	T15	T60
Dog	a	16.03	13.41	15.26
	b	17.03	16.58	15.83
	c	14.23	11.48	12.51
	d	14.59		
	e	13.27		
	f	15.94	13.75	13.91
	Mean	15.2	13.8	14.4
	SD	1.4	2.1	1.5
